# Evidence for Kinetic Limitations as a Controlling Factor of Ge Pyramid Formation: a Study of Structural Features of Ge/Si(001) Wetting Layer Formed by Ge Deposition at Room Temperature Followed by Annealing at 600 °C

**DOI:** 10.1186/s11671-015-0994-0

**Published:** 2015-07-16

**Authors:** Mikhail S. Storozhevykh, Larisa V. Arapkina, Vladimir A. Yuryev

**Affiliations:** A. M. Prokhorov General Physics Institute of the Russian Academy of Sciences, 38 Vavilov Street, Moscow, 119991 Russia; Technopark of GPI RAS, 38 Vavilov Street, Moscow, 119991 Russia

**Keywords:** Ge/Si(001) clusters, Kinetic-controlled growth, Equilibrium structure

## Abstract

The article presents an experimental study of an issue of whether the formation of arrays of Ge quantum dots on the Si(001) surface is an equilibrium process or it is kinetically controlled. We deposited Ge on Si(001) at the room temperature and explored crystallization of the disordered Ge film as a result of annealing at 600 °C. The experiment has demonstrated that the Ge/Si(001) film formed in the conditions of an isolated system consists of the standard patched wetting layer and large droplike clusters of Ge rather than of huts or domes which appear when a film is grown in a flux of Ge atoms arriving on its surface. We conclude that the growth of the pyramids appearing at temperatures greater than 600 °C is controlled by kinetics rather than thermodynamic equilibrium whereas the wetting layer is an equilibrium structure.

***PACS:*** Primary 68.37.Ef; 68.55.Ac; 68.65.Hb; 81.07.Ta; 81.16.Dn

## Background

The issue of whether formation of arrays of Ge quantum dots on the Si(001) surface is an equilibrium driven or kinetically controlled process has not been solved since the very discovery of coherent GeSi and Ge islands by Eaglesham and Cerullo [1] and Mo et al. [2]. Numerous articles supported the model of kinetically driven growth of Ge clusters whereas others gave theoretical and experimental evidences of the equilibrium nature of Ge quantum dot arrays (see, e.g., refs. [3–12]). In 1999, a detailed analysis of this long-standing (even at that time) issue was made by Shchukin and Bimberd in their now already classical review cited in ref. [13] which covered a wide selection of various heteroepitaxial systems including Ge/Si(001) epitaxial films with Ge clusters. Additional discussions of this issue can be found in a brief review section included to ref. [14] or in our recent review article appeared in ref. [15].

This article presents an experimental study of this issue. We deposited Ge on Si(001) at the room temperature and explored crystallization of the disordered Ge film as a result of a thermal treatment. This experiment, originally conceived as purely technological, gave somewhat unexpected results. First of all, it demonstrated that the Ge/Si(001) film formed in the conditions of a closed system consists of the usual patched wetting layer (WL) and large droplike clusters of Ge rather than huts or domes which appear when a film is deposited in a flux of Ge atoms arriving on its surface. Additionally, we observed nucleation of the 2×1 reconstructed phase (i.e., the ordered one) in the disordered Ge film deposited at the room temperature. And finally, we detected a mixture of *c*(4×2) and *p*(2×2) reconstructions on the surface of the resultant Ge film annealed at high temperature (600 °C) whereas the simultaneous presence of both these structures in comparable proportions on wetting layer patches is a distinctive feature of the low-temperature mode of the wetting layer growth (<600 °C) [16].

Now, we proceed to the detailed presentation of the obtained results.^1^

## Methods

### Equipment, techniques, and samples

Experiments were carried out using a specially built setup consisting of a ultrahigh-vacuum (UHV) molecular-beam epitaxy (MBE) chamber (Riber EVA 32) equipped with a RH20 reflected high-energy electron diffraction (RHED) unit (Staib Instruments) and connected with a UHV STM chamber (GPI 300) [17–19].

The Ge deposition rate and the Ge coverage (*h*_Ge_) were measured by a graduated in advance film thickness monitor (Inficon Leybold-Heraeus XTC 751-001-G1) with a quartz sensor installed at the MBE chamber. Samples were heated from the rear side by radiators of tantalum. The temperature was monitored with a tungsten-rhenium thermocouple mounted in the vacuum nea the rear side of the samples and *in situ* graduated beforehand against a specialized pyrometer (IMPAC IS 12-Si) which measured the Si sample temperature *T* through a chamber window with an accuracy of ±(0.003 *T* [°C] + 1) °C. The atmosphere composition in the MBE chamber was monitored using a mass-spectrometer residual gas analyzer (SRS RGA-200) before and during the process. Additional details concerning the used equipment can be found, e.g., in refs. [14, 17, 18].

Details of the pre-growth treatments of Si wafers, which included wet chemical etching and oxide removal by short high-temperature annealing (*T*∼ 900 °C), can be found in our previous articles [20–22]. Ge films (*h*_Ge_=7 Å) were deposited at the rate of 0.15 Å/s directly on the clean Si(001) surface at the room temperature. Then the samples were heated to 600 °C at the maximum rate achievable for the used infrared heaters (0.24 °C/s),^2^ annealed at this temperature for 5 min and cooled to the room temperature at the quenching mode [23] at the rate of 0.4 °C/s. Afterwards, the samples were moved into the STM chamber for the structural analysis. STM images were processed using the WSxM software [24]. Reflected high-energy electron diffraction (RHEED) patterns were monitored on a screen and recorded with a video camera during the whole cycle of Ge film deposition and heat treatment.

## Results and Discussion

### A Structure of the Initial Ge Film

**Fig. 1:**
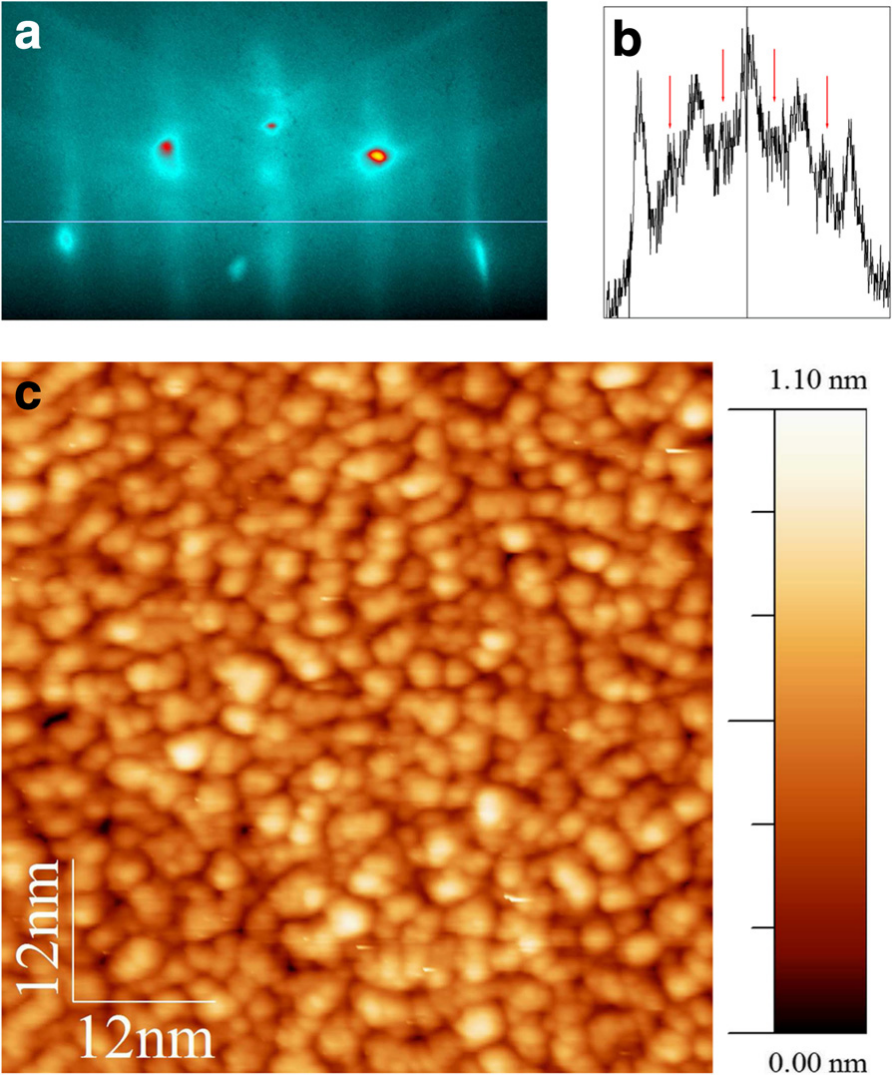
A RHEED pattern and an STM image of the initial Ge film. **a** The RHEED pattern obtained at the room temperature after Ge film deposition before sample heating (the effective thickness *h*_Ge_ = 7 Å; [110] azimuth, *E* = 10 keV) and **b** its profile taken along the *light line* in the panel (**a**); *arrows* in the panel (**b**) indicate the weak ½-reflexes virtually unobservable at the fluorescent screen during the experiments but visible in the panel (**a**) which demonstrate that the 2 × 1 structure occupies a minor part of the film surface area. **c** The STM image demonstrates a grainy disordered structure of the film

When Ge is deposited on the Si(001) surface at the room temperature, the 2×1 RHEED pattern of Si(001) evolves into the 1×1 one with the gradually decaying 1/2-streaks as the thickness of the deposited Ge film (*h*_Ge_) increases. However, even at *h*_Ge_=7 Å, the 1/2-reflexes are still observable in the diffraction pattern (Fig. 1a, b) that is a direct indication of the presence of the 2×1 reconstructed domains of the film surface which occupy a part of its area. At the same time, the visible diffuse scattering of electrons and widening of the streaks indicates that the film is significantly disordered. So, we should conclude that an ordered 2×1 reconstructed crystalline phase, although occupying a minor part of the surface, forms in the disordered Ge film deposited on Si(001) at the room temperature.

STM images obtained from the as-deposited Ge film demonstrate a highly disordered granular structure (Fig. 1c). The film thickness reaches 1 nm due to its porosity. We failed to resolve the 2×1 reconstructed islands or areas. However, this does not allow one to state that such reconstructed domains are absent since the atomic resolution necessary for their observation was not reached at the mainly disordered surface of the granular (and porous) Ge film. The RHEED patterns themselves demonstrating such reconstruction are a sufficient evidence for its presence on the surface.

### A Structure of the Ge/Si(001) Film after Annealing

#### The Wetting Layer

**Fig. 2:**
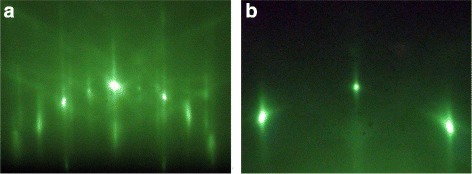
RHEED patterns obtained at room temperature after sample cooling. *E* = 10 keV; **a** [110] and **b** [100] azimuths; streaks of the 2 × 1 structure and 3D reflexes (discontinuous streaks) are observed

RHEED patterns of the Ge layer drastically change as a result of sample annealing (Fig. 2): streaks of the 2×1 structure as well as 3D features are now observed. The 3D pattern becomes visible during sample heating at the temperature some higher than 500 °C; the pronounced 2×1 structure appears, as it has already been shown previously [15, 25], during cooling at the temperature less then 600 °C. So, we can conclude that the disordered Ge film deposited at the room temperature on Si(001) transforms as a result of annealing at 600 °C into the ordered 2×1 reconstructed Ge layer.

**Fig. 3:**
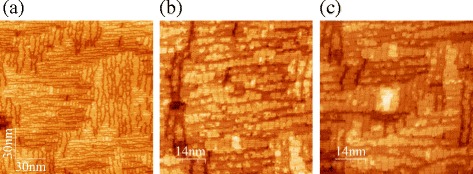
STM micrographs of the annealed sample obtained at different points on the surface. A usual *M* ×*N* reconstruction composed by *p*(2 × 2) and *c*(4 × 2) reconstructed patches is observed (the *p*(2 × 2) reconstruction is seen as in-phase zigzags, the *c*(4 × 2) one is seen as anti-phase zigzags). **a**–**c** The images were obtained at different points on a sample

**Fig. 4:**
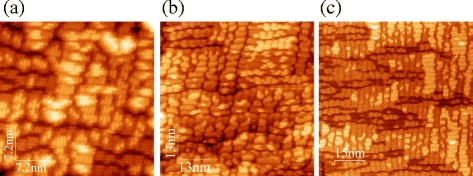
STM images of the Ge/Si(001) wetting layers grown at different conditions. **a** *T*_gr_ = 360 °C, *h*_Ge_ = 4 Å; **b** *T*_gr_ = 600 °C, *h*_Ge_ = 6 Å; **c** *T*_gr_ = 650 °C, *h*_Ge_ = 4 Å

Furthermore, STM images of the resultant surface demonstrate typical pictures of the *M*×*N* reconstruction (Fig. 3): the surface is composed by patches bounded by the dimer-row vacancies and dimer vacancy lines [26–30]. There is no visible difference between this wetting layer, obtained as a result of annealing of the disordered Ge film, and wetting layers grown by molecular beam epitaxy at different conditions, both in low-temperature and high-temperature modes (Fig. 4); the only specific feature of this wetting layer is that, being formed at high temperature, it contains both *p*(2×2) and *c*(4×2) structures whereas only (or nearly only) the latter one is observed on the wetting layers deposited at high temperatures and a mixture of the two reconstructions is a characteristic feature of the wetting layers deposited at low temperatures [15, 25, 31, 32]. But in general this minor peculiarity does not seem to be very significant in this case.

Thus, we can conclude from the above that formation of the Ge/Si(001) wetting layer is controlled by system evolution to thermodynamic equilibrium, i.e., it is an equilibrium system.

#### Ge Hillocks

**Fig. 5:**
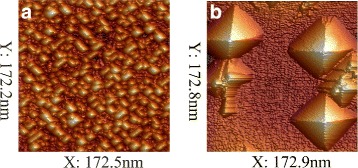
STM micrographs of Ge huts forming at different conditions. **a** *T*_gr_ = 360 °C, *h*_Ge_ = 7 Å; **b** *T*_gr_ = 600 °C, *h*_Ge_ = 6 Å

**Fig. 6:**
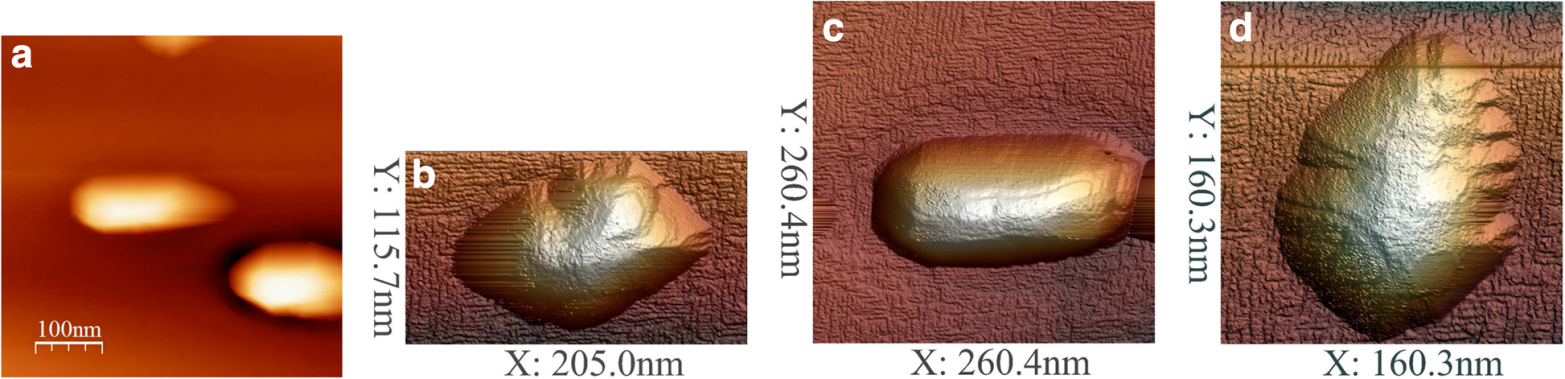
**a**–**d** STM images of Ge clusters observed on the wetting layer after annealing at 600 °C of the Ge film deposited at the room temperature. *h*_Ge_ = 7 Å

The thickness of the Ge/Si(001) wetting layer is known to be equal to four monolayers that is a little greater than 5.5 Å. We deposited 7 Å of Ge. Usually, if Ge is deposited at low temperatures (say, e.g., at *T*_gr_ = 360 °C), at such coverages, the excess Ge atoms form the well-recognizable structure of the hut array (Fig. 5a) [2, 15, 17]; if Ge is deposited at 600 °C (or at the high-temperature mode), the excess Ge starts to gather into cluster arrays at even less coverages and forms pyramids in which split edges are often observed (Fig. 5b) [32–35]. The question is where the excess Ge deposited at the room temperature flows during the heat treatment at 600 °C. As we have observed, at these treatments, Ge forms neither hut arrays nor pyramids or domes, but it is gathered into large partially faceted droplike clusters (Fig. 6). The lateral dimensions of these oval hillocks vary from about 100 to about 200 nm, their heights vary from nearly 10 to more than 20 nm, and their number density makes (1.2–1.3) ×10^9^ cm ^−2^. “Pelerines” of multiple incomplete facets seen around apexes (Fig. 6b) and indicating that these clusters grow from tops to bottoms [33], as well as split edges (Fig. 6b, d), demonstrate that they have some common features with the pyramids growing at the same temperature during the molecular beam epitaxy (Fig. 5b).

The presented observation allows us to definitely state that no huts (or domes) appear if a Ge/Si(001) film forms moving to equilibrium as a closed system (or, in fact, as an isolated system since outflow and radiative inflow of energy are balanced). Pyramid arrays (no wedges arise at high temperatures [32]) emerge only as a result of a process requiring Ge atoms to flow on the surface. So, this experiment unambiguously demonstrates that at least the growth of the high-temperature pyramids appearing at temperatures greater than 600 °C is controlled by kinetics rather than evolution to thermodynamic equilibrium; equilibrium clusters are the oval ones.

Reasoning which allows us to come to this conclusion is very simple. The question is about the driving force of Ge cluster formation: if it is kinetics (random migration of atoms and their interactions) or thermodynamics (evolution of a system to equilibrium). The Ge/Si(001) isolated system comes to a thermodynamic equilibrium state for the given temperature as a result of isothermal annealing [36]. If the pyramids would be equilibrium (or thermodynamically driven) structures, they would appear as a result of annealing; or in other words, if they do not appear they are not equilibrium at high-temperature growth. We can conclude now that at high temperatures pyramids arise due to kinetics rather than thermodynamics, and thermodynamics would give rise to the oval clusters instead of the pyramids.

It should be noted that we consider the oval mounds as equilibrium structures (or maybe close to equilibrium, since this does not matter for our conclusions) despite that the annealing time seems too short. We believe that this conclusion is correct because the process, which first would result in appearance of large oval mounds on WL and then in their dissolution or reformation into some other structures, seems to be very unlikely in a closed system. Also we can hardly assume that the oval cluster formation is constrained by some other kinetic processes, different from those constraining the pyramid formation during MBE, because the closed system that we consider (which is the isolated system at the phase of isothermal annealing) should evolve to its thermodynamic equilibrium, and even if the oval mounds are not yet completely equilibrium formations, they are certainly on this way and they likely may become larger or differently faceted in equilibrium but they may not become pyramids similar to those appearing as a result of MBE.

Now let us consider in a few words the pyramid formation in the process of MBE. There are two competing kinetic processes on WL during MBE at the temperature of the described experiment: a process of a pyramid formation near a place of Ge atom “landing” on WL requiring a small migration length and a process of a long-distance migration of the “landed” Ge atoms along the WL surface resulting in appearance of oval clusters (whose number density is only about 10^9^ cm ^−2^). Probably, the latter process would be favorable at very low Ge deposition rates, approaching zero, when the system resembles the closed one and local supersaturation does not appear or has enough time to disappear due to adatom migration. At practical deposition rates, local supersaturation rises rapidly, Ge adatoms are incorporated by WL; WL patches, whose upper layers are unstrained [15, 25, 31], can no longer consume Ge, and the strain would begin to grow unless Ge atoms start to form pyramids on patches as described by our model presented in ref. [31]. So, kinetics governs the processes of nucleation and growth of the high-temperature pyramids; otherwise, the excess Ge adatoms would be distributed into the observed oval clusters as it is prescribed by thermodynamics.

We should note also that unlike SiGe islands grown by Ge diffusion from a local source [37, 38] at the temperatures close or equal to the temperature of the discussed experiment, which also have oval shapes although some different from those described in this article, the oval mounds described by us evolve to equilibrium with the wetting layer. The islands studied in refs. [37, 38] arise and grow in a permanent flux of adatoms directionally migrating along the wetting layer from the Ge stripe (the source of Ge) to the Si surface free of Ge. Consequently, they never come to equilibrium with the wetting layer (although they grow under the isothermal annealing) and their formation is controlled by kinetic processes, i.e., by diffusion and capture of diffusing atoms.

## Conclusions

In conclusion of the article, we would like to emphasize its main statements.

First, we conclude that an ordered 2×1 reconstructed crystalline phase forms in the disordered Ge film during its deposition on the Si(001) surface at the room temperature. Second, as a result of annealing at 600 °C, the disordered Ge film deposited at the room temperature on Si(001) transforms into the ordered 2×1 reconstructed Ge wetting layer. Third, the resultant wetting layer surface is *M*×*N* reconstructed, i.e., it consists of patches bounded by the dimer-row vacancies and dimer vacancy lines; apexes of the patches are formed by both *p*(2×2) and *c*(4×2) structures. Fourth, huts (pyramids or wedges) or domes do not emerge if a Ge/Si(001) film is formed in a closed system by annealing at 600 °C of a Ge layer deposited on Si(001) at the room temperature; the excess Ge atoms from the initial Ge film are gathered into large partially faceted oval clusters.

And finally, we have demonstrated that at least the growth of the pyramids appearing at temperatures greater than 600 °C is controlled by kinetics rather than evolution to thermodynamic equilibrium whereas the wetting layer is an equilibrium structure. Consequently, we can conclude that these nonequilibrium, kinetically controlled, Ge quantum dots grow on the equilibrium, thermodynamically controlled, Ge/Si(001) wetting layer. Thus, the wetting layer could determine only the places of their nucleation and maybe the structure of their nuclei but cannot control their growth process; this inference completely agrees with the hut nucleation and growth scenarios proposed in our previous publications [15, 25, 31].

## Endnotes

^1^ A preprint of the current article is cited in Ref. [39].

^2^ The heating process does not seem to be really adiabatic: its rate was insufficiently high and we observed changes in the surface structure that means that some changes in entropy took place. However, we performed heating as rapidly as we could to minimize its effect on the Ge layer.

## Abbreviations

MBE: molecular beam epitaxy
RHEED: reflected high-energy electron diffraction
WL: wetting layer
